# High-Grade Undifferentiated Pleomorphic Sarcoma of the Tongue: A Case Report

**DOI:** 10.7759/cureus.93850

**Published:** 2025-10-04

**Authors:** Iqrah Muhammad, Carolina Jaramillo, Raul Puente-Vallejo, Cristina Núñez, Zelma Paz

**Affiliations:** 1 College of Health Sciences, University San Francisco de Quito, Quito, ECU; 2 Radiation Oncology Department, Solca Nucleo de Quito Hospital, Quito, ECU; 3 Radiation Oncology Department, Hospital Metropolitano Quito, Quito, ECU; 4 Pathology Department, Solca Nucleo de Quito Hospital, Quito, ECU

**Keywords:** malignant fibrous histiocytoma, radiotherapy (rt), sarcoma, tongue, undifferentiated pleomorphic sarcoma (ups)

## Abstract

Undifferentiated pleomorphic sarcoma (UPS) is a rare entity that mainly affects the extremities and is uncommon in the head and neck region. Arising from mesenchymal cells with complex genomic alterations, its typical clinical presentation involves a rapidly growing mass in elderly patients. Although surgical resection is the main treatment, high rates of recurrence and metastasis are usually present, requiring radiotherapy or chemotherapy as complementary therapies.

We present the case of a 75-year-old female patient with a tongue mass, initially diagnosed as a fibrous tumor and subsequently as a high-grade pleomorphic undifferentiated sarcoma of the tongue. After an initial hemiglossectomy and a second surgery with wide excision, reconstruction, and radiotherapy, no evidence of residual disease was observed at follow-up. Despite developing xerostomia and dysarthria as late side effects of treatment, the patient showed a favorable response to treatment, with regular check-ups revealing no tumor activity until 24 months after treatment. This case highlights the importance of a multidisciplinary approach in the management of tongue sarcoma, showing that a combination of surgery, radiotherapy, and close follow-up can result in the successful control of this pathology, even in highly aggressive cases.

## Introduction

Pleomorphic sarcoma of the tongue is an uncommon and poorly documented tumor in medical literature [1]. It is characterized by aggressive behavior, high recurrence rates, and significant therapeutic challenges due to the complex anatomy of the head and neck and the absence of standardized treatment guidelines [2]. Fewer than 30 cases have been reported worldwide [3,4], and to our knowledge, none have been reported in Latin America. Reporting additional cases is therefore valuable to better highlight the importance of a multidisciplinary approach and its management [5]. We present the case of a 75-year-old patient diagnosed with undifferentiated pleomorphic sarcoma of the tongue, with a favorable evolution without relapse up to 24 months, despite the size, tumor subtype, associated poor prognostic factors, and treatment delays. This article will provide a detailed analysis of a clinical case of pleomorphic sarcoma of the tongue, examining the current status of the guidelines for the diagnosis and management of this type of tumor.

## Case presentation

We present a 75-year-old woman with no relevant clinical or pathological history. She had a history of four months of asthenia and difficulty in speech articulation, associated with the appearance of a progressively growing mass on the left border of the tongue. During the initial evaluation in a first medical institution without previous imaging studies, a partial left glossectomy with resection of left cervical lymph nodes level III was performed. The initial histopathological study suggested a fibrous tumor. Owing to delays within the public health system, the patient was not referred to a second institution until seven weeks after the surgical procedure. On physical examination, a 1.5 cm surgical scar was noted on the anterior third of the left lateral tongue border, without evidence of macroscopic tumor activity. In the neck, a scar of the left lymph node resection was identified, with no presence of locoregional adenopathies.

**Figure 1:**
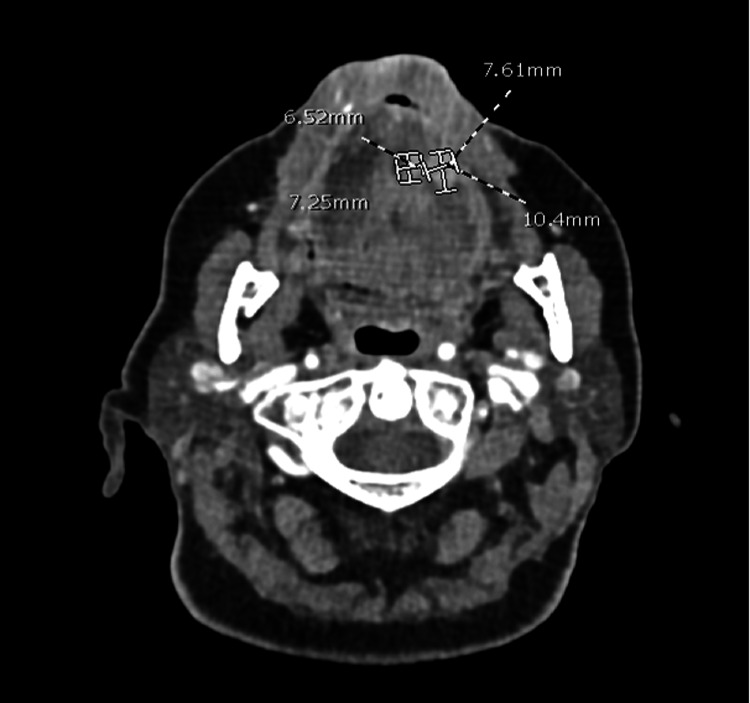
CT of the face, axial view The image shows postcontrast heterogeneous enhancement in the left aspect of the genioglossus muscle, as well as two nodular images with slight homogeneous postcontrast enhancement of 7.2 x 6.5 mm and 10.4 x 7.6 mm in their anteroposterior and transverse diameters, suggesting post-surgical tumor activity. CT: Contrasted computed tomography

The contrasted computed tomography (CT) of the face showed thickening of the left aspect of the genioglossus muscle, with slight heterogeneous enhancement postcontrast. Additionally, two nodular lesions with slight homogeneous postcontrast enhancement were identified, measuring 7.2 mm anteroposterior × 6.5 mm transverse, and 10.4 mm anteroposterior × 7.6 mm transverse, respectively. The larger lesion was in contact with the edge of the genioglossus muscle without an adequate plane of separation, suggesting post-surgical tumor activity, as shown in Figure 1.

**Figure 2:**
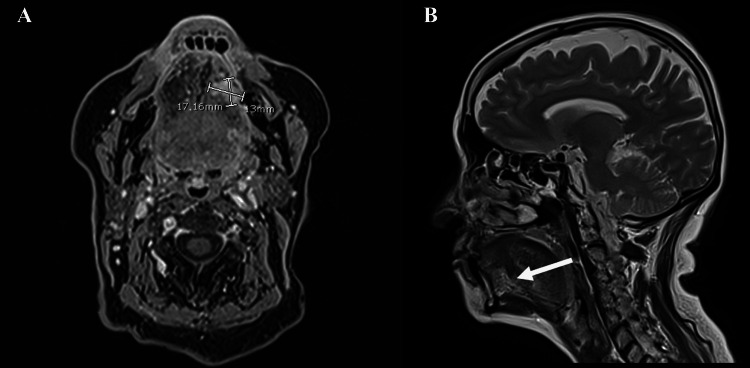
MRI of head and neck: (A) T1 sequence contrasted in the axial plane and (B) T2 sequence in the sagittal plane Image (A) shows an axial MRI scan revealing a poorly defined lesion on the left lateral border of the tongue, with intense post-contrast enhancement. The lesion measures 11 × 13 × 17 mm in the longitudinal, anteroposterior, and transverse axes, respectively. Image (B) shows the same lesion in the sagittal plane (indicated by the white arrow). MRI: Magnetic resonance imaging

Contrast CT of the neck and thorax revealed no evidence of lymphadenopathy or distant metastatic lesions. Magnetic resonance imaging (MRI) showed a poorly defined lesion on the left lateral border of the tongue with intense postcontrast enhancement measuring 11 x 13 x 17 mm in the longitudinal, anteroposterior, and transverse axes, respectively. The lesion involved the left hyoglossus and genioglossus muscles, including longitudinal fibers and extending to the lingual septum (Figure 2).

**Figure 3:**
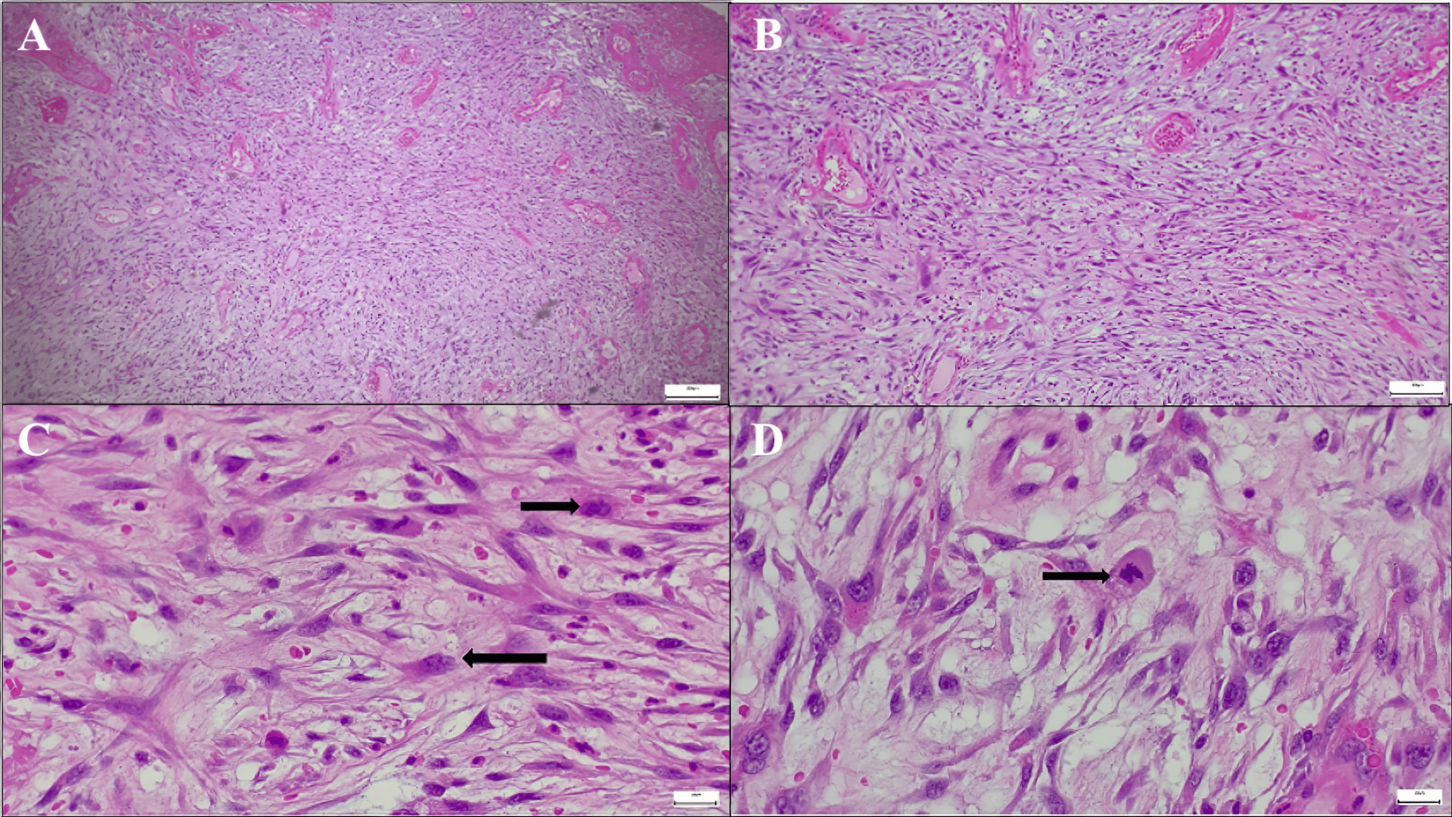
Microscopic image with H&E staining of pleomorphic sarcoma with different magnifications The image shows: (A) an H&E stain at 5× magnification revealing a hypercellular neoplasm composed of spindle cells arranged in a disordered pattern; (B) an H&E stain at 10× magnification showing a spindle cell neoplasm with pleomorphic nuclei and disorganized architecture; (C) and (D) H&E stains at 40× magnification highlighting spindle cells with pleomorphic nuclei and atypical mitotic figures (indicated by black arrows). H&E: hematoxylin-eosin

**Figure 4:**
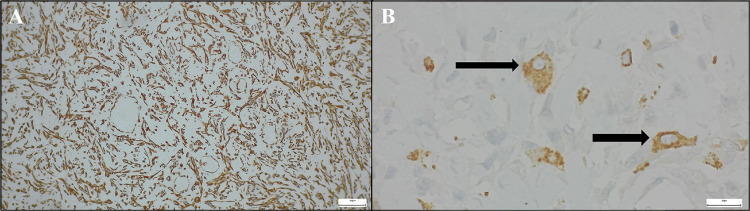
Microscopic images with immunohistochemical staining of pleomorphic sarcoma: (A) vimentin stain and (B) CD68 stain The image shows: (A) an immunohistochemistry study at 20× magnification using vimentin, showing positive staining consistent with a sarcomatous neoplasm; (B) an immunohistochemistry study at 40× magnification using CD68, with positive staining in neoplastic cells (indicated by black arrows).

Review of the pathologic findings from the first surgery indicated an initial lesion measuring 4.5 x 4 x 3 cm, with a histopathologic diagnosis of high-grade pleomorphic undifferentiated sarcoma of the tongue. Tumor involvement of the anterior margin and absence of metastasis in the 15 nodes were analyzed (Figures 3, 4).

Based on the findings, the patient underwent a left hemiglossectomy 19 weeks after the initial procedure. A wide excision was performed with 1 cm margins to achieve resection with negative margins (R0). To restore form and critical function (speech and swallowing) after the extensive resection in a highly functional area, reconstruction was carried out using a left radial forearm free flap and fasciocutaneous flaps. A tracheostomy and gastrostomy were also performed. A specimen measuring 6 × 5 × 3 cm was resected, and subsequent pathological analysis revealed no residual tumor. A follow-up CT scan of the neck and thorax performed 11 weeks postoperatively showed no abnormal contrast enhancement or suspicious cervical lymphadenopathy.

**Figure 5:**
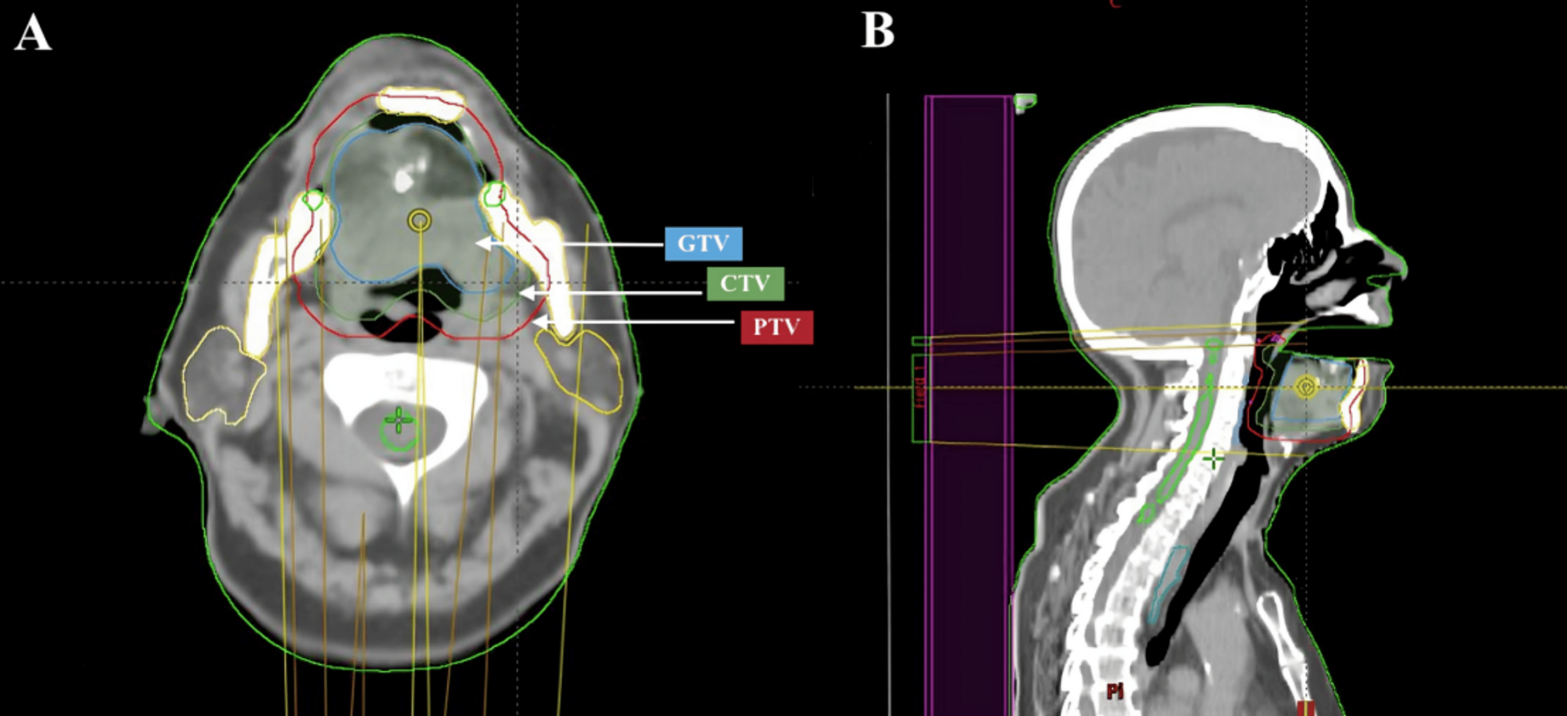
Treatment target volume delineation axial (A) and sagittal (B) The figure illustrates the delineation of the target volumes, with white arrows indicating the structure's GTV, CTV, and PTV. GTV: gross tumor volume; CTV: clinical target volume; PTV: planning target volume

**Figure 6:**
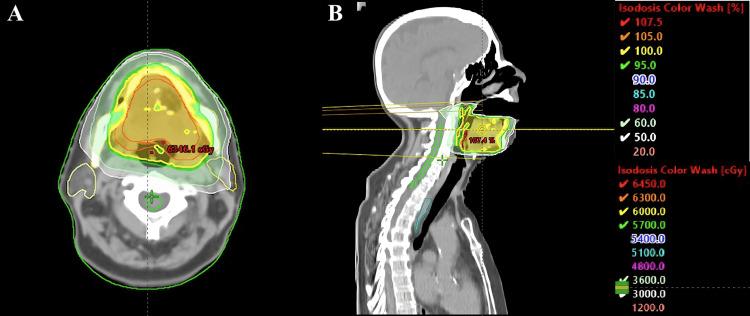
Treatment planning dose distribution: axial (A) and sagittal (B) planes The figure shows the treatment volume with dose distribution, with yellow representing 60Gy.

Based on the histopathological diagnosis of a high-grade sarcoma and the patient’s history of an initial inadvertent excision without a preoperative diagnosis of sarcoma, adjuvant radiotherapy (RT) was added following the wide excision. Treatment was delivered using volumetric modulated arc therapy (VMAT), with a total dose of 60 Gy delivered in 30 fractions over 6 weeks, focusing on the tongue region. The total prescribed dose was defined according to the negative surgical margins obtained during the wide excision [2,6]. Treatment contouring followed the departmental protocol, which included both non-contrast and contrast-enhanced CT scans with 3 mm slice thickness, in accordance with the institution’s standard procedures and integrating guideline recommendations [7,8]. The gross tumor volume (GTV) was defined using surgical reports, then expanded by 5 mm to create the clinical target volume (CTV), and an additional 5 mm margin was applied to generate the planning target volume (PTV). Online image-guided radiation therapy (IGRT) was performed during each treatment session using cone-beam CT (CBCT) to ensure precise patient positioning and alignment with the planning CT. The treatment volume is illustrated in Figures 5, 6.

**Figure 7:**
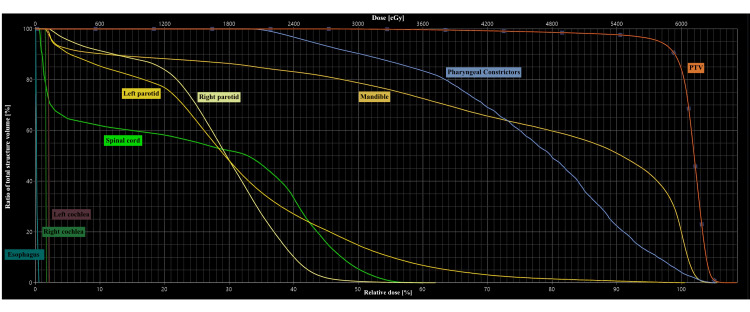
Treatment dose–volume histogram (DVH) The figure shows the DVH curves for the target volume PTV and the organs at risk. The structures are color-coded as indicated in the figure. DVH: treatment dose–volume histogram; PTV: planning target volume

The dose volume histogram is shown in Figure 7, and the dosimetric results are summarized in Table 1.

**Table 1:** Target dose and organs at risk constraints achieved in radiotherapy treatment PTV: planning target volume

Plan			Tongue		
Total Dose			6000.0 cGy		
Structure Name	Clinical goal summary		1	0	13
PTV 60Gy	P1	D 98.0 % ≥ 95.0 %	87.70 %		
	P1	D 50.0 % ≤ 102.0 %	101.98 %		
	P1	D 50.0 % ≥ 100.0 %	101.98 %		
	P1	Dmax ≤ 110.0 %	108.66 %		
	P1	D 2.0 % ≤ 107.5 %	104.73 %		
Right cochlea	P3	Dmax ≤ 5000 cGy	104.89 cGy		
Left cochlea	P3	Dmax ≤ 5000 cGy	129.92 cGy		
Esophagus	P3	V 6000 cGy < 7.0 %	0.00 %		
	P3	V 7000 cGy < 0.1 cm3	0.00 cm3		
Mandible	P3	Dmax ≤ 7000 cGy	6337.55 cGy		
Spinal cord	P2	Dmax ≤ 4500 cGy	3597.18 cGy		
Right parotid	P3	V 3000 cGy < 50.0 %	0.54%		
	P3	Dmedia ≤ 2500 cGy	1703.34 cGy		
Left parotid	P3	V 3000 cGy < 50.0 %	14.79 %		
	P3	Dmedia ≤ 2500 cGy	1874.10 cGy		
Pharynx constrictor	P3	Dmedia < 5000 cGy	4599.16 cGy		

Follow-up imaging performed 10 weeks after the completion of radiotherapy demonstrated no evidence of residual or recurrent tumor activity. Both acute and late toxicities were evaluated and graded according to the Common Terminology Criteria for Adverse Events (CTCAE) Version 5.0. Acute toxicity was limited to grade 2 mucositis, which was managed symptomatically with analgesics, supportive care, and nutritional assistance, without the need for prophylactic medications, allowing treatment to continue uninterrupted. Late toxicities included grade 1 chronic xerostomia and grade 2 dysarthria.

Since then, the patient has been closely monitored with clinical evaluations every three months, showing no evidence of disease recurrence. Follow-up included CT scans of the head and neck, face, chest, abdomen, and pelvis, as well as ultrasound examinations of the neck and abdomen, to exclude local or distant disease. Positron emission tomography/computed tomography (PET/CT) imaging was not performed due to limited availability within the institution. At 24 months post RT treatment, the patient remains disease-free, with no clinical or radiological evidence of recurrence.

## Discussion

Sarcomas are malignant neoplasms that develop from transformed mesenchymal cells and can arise in various areas of the human body from different tissues such as bone and soft tissues [9]. Undifferentiated pleomorphic sarcoma (UPS), formerly known as malignant fibrous histiocytoma, stands out as a high-grade and very aggressive sarcoma within the group of soft tissue sarcomas [10], which includes various types of neoplasms arising from muscle, adipose tissue, connective tissue, blood vessels, lymphatics, and peripheral nerves [2]. The 2020 World Health Organization (WHO) classification of soft tissue sarcomas incorporates UPS under malignant tumors of uncertain differentiation [11]. The pathogenic mechanisms of UPS are complex and unclear. It is known that UPS tumorgenesis initiates with cells in which signaling pathways, such as Hedgehog, Notch, and Hippo, are activated, conferring them the capacity for self-renewal and growth [12]. In addition, exposure to ionizing radiation has been linked as a risk factor [13].

Sarcomas are rare, accounting for 1% of adult and 15% of pediatric malignancies [2]. Among them, UPS occurs predominantly in adults, more often in males, with risk increasing after the sixth decade [1,10]. They can arise in various anatomical sites, most commonly the extremities (43%). With head and neck involvement being uncommon (3-10%) and tongue localization extremely rare (<30 cases reported) [1-4]. They typically present as rapidly growing, painless masses, causing functional symptoms, such as dysarthria or swallowing difficulties in the oral cavity, which may later lead to respiratory problems as the tumor grows [1]. Imaging, such as CT and MRI, and histopathology are crucial for diagnosis, but findings are often nonspecific [4,14]. Immunohistochemistry primarily aids in excluding other lineages, with tumor cells typically showing strong vimentin expression and cytoplasmic CD68 positivity (a histiocyte marker), while consistently negative for other lineage-specific markers, making UPS a diagnosis of exclusion [10,15].

Treatment of head and neck soft tissue sarcomas depends on histology, location, stage, tumor size, and patient age. Due to their rarity, management follows sarcoma guidelines from other anatomic sites. Standard treatment is en bloc surgical excision with wide margins to achieve microscopically negative (R0) margins [2], which can be challenging in this region due to complex anatomy, the proximity of vitally important neurovascular structures, functional alterations related to breathing, swallowing, and speech, as well as cosmetic considerations [16]. RT and chemotherapy (QT) play complementary roles in sarcoma management, depending on disease stage and risk factors, and a multidisciplinary approach is essential to define their optimal combination or sequence. RT is recommended for patients at high risk of local recurrence, including those with positive or close (<1 mm) margins, or involvement of bone, major vessels, or nerves [2]. Although RT improves local control, it does not significantly affect the development of distant metastases (DM) or overall survival (OS) [17]. RT can be delivered pre- or postoperatively, definitively in unresectable or oligometastatic disease, or palliatively [17,18]. QT is mainly indicated for advanced or high-risk tumors, with anthracycline regimens as the first line [2]. Targeted therapies, such as pazopanib and immune checkpoint inhibitors (pembrolizumab, nivolumab, ipilimumab), are under investigation [19,20], while catheter-directed approaches may be considered for unresectable disease or patients unfit for surgery [2].

UPS are high-grade lesions with local recurrence rates of 19-31% and metastatic rates of 31-35% [4]. Lymphatic metastases are uncommon, with the lung being the most frequent site of distant dissemination (90%), followed by bone (8%) and liver (1%) [4]. Both local recurrence and distant metastases typically occur within 12-24 months after diagnosis [4]. The 5-year survival rate for UPS in the head and neck is 50-60%, compared with 73% in other locations [21]. General predictors of poor prognosis include male sex, advanced age, and the presence of metastases at diagnosis, while post-surgical factors include age over 70 years, positive surgical margins, and tumors larger than 5 cm [22].

This case illustrates an uncommon presentation of UPS on the lateral border of the tongue, where the most notable clinical features were dysarthria and swallowing difficulties. Differentiation from more common oral malignancies, such as squamous cell carcinoma, remains challenging due to the lack of major distinctive clinical signs. The patient initially underwent an unplanned excision at a non-specialized center, where limited access to immunohistochemical testing led to misclassification as a fibrous tumor. Such initial management increased the risk of recurrence and potentially compromised overall outcomes. Referral through the public health system introduced further delays, and upon evaluation at a specialized cancer center, the diagnosis was corrected to UPS, revealing positive margins that required a second, more extensive surgery in a complex anatomical region, resulting in additional morbidity. Radiotherapy was initiated 11 weeks after this second intervention due to delays in control imaging and a long waiting list for consultation with the radiotherapy team. Delivered using advanced techniques, radiotherapy played a crucial role in achieving effective local disease control, particularly given the high-risk features, compromised margins, and prior unplanned procedures. At 24 months of follow-up, the patient remains free of local recurrence and distant metastases.

## Conclusions

UPS of the tongue is a rare tumor with no standardized therapeutic protocol. This diagnostic possibility should be considered when evaluating tongue lesions. In our case, despite multiple delays in diagnosis and management, effective disease control was achieved, with advanced radiotherapy techniques playing a pivotal role in ensuring local control. The case highlights the importance of adequate multidisciplinary evaluation prior to treatment and underscores the urgent need to strengthen public health systems in low-resource settings, where timely access to histopathology, imaging, and radiotherapy at the initial center could have prevented misdiagnosis, reduced delays, preserved information during referrals, and enabled coordinated treatment, factors critical for optimizing disease control in tumors with inherently poor prognosis.
